# The mediational role of physical activity, social contact and stroke on the association between age, education, employment and dementia in an Asian older adult population

**DOI:** 10.1186/s12888-017-1272-8

**Published:** 2017-03-20

**Authors:** Edimansyah Abdin, Siow Ann Chong, Chao Xu Peh, Janhavi Ajit Vaingankar, Boon Yiang Chua, Swapna Verma, Anitha Jeyagurunathan, Saleha Shafie, Mythily Subramaniam

**Affiliations:** 1 Research Division, Institute of Mental Health, Buangkok Green Medical Park, 10 Buangkok View, Singapore, 539747 Singapore; 2 Early Psychosis Intervention Programme, Institute of Mental Health, Buangkok Green Medical Park, 10 Buangkok View, Singapore, 539747 Singapore

**Keywords:** Dementia, Population-based, Elderly, Mediation, Moderator, Exercise, Social contact, Stroke

## Abstract

**Background:**

Our study aimed to investigate the pathways by which socio-demographic factors, modifiable health and lifestyle risk factors influence each other, and subsequently, lead to dementia.

**Methods:**

We used data from the Well-being of the Singapore Elderly study, a nationally representative survey of the older adult population aged 60 years and above in Singapore. Dementia diagnosis was established using 10/66 dementia criteria. Structural equation modelling (SEM) without latent variable was applied to confirm the hypothesized model.

**Results:**

The results of SEM supported the hypothesized model (*χ* ^2^ = 14.999, *df* = 10, *p* = 0.132). The final model showed that those aged 75–84 years and 85 years and over (vs. 60–74 years), having no formal education, who had completed primary or secondary education (vs. completed tertiary), who were homemakers and retired (vs. paid work), and with a history of stroke were directly associated with higher odds of having dementia, while those who had more frequent contact with friends and neighbors as well as being physically active were directly associated with lower odds of having dementia diagnosis. The study also found that physical activity, more frequent contact with friends and stroke played a significant role as mediators in these relationships. The overall pathways model explained 57.7% of the variance in dementia.

**Conclusion:**

Our results suggest that physical activity, social contact and stroke were potential mediators in the relationship between age, education, employment and dementia. Intervention programmes focusing on physical activity such as exercise and social contact may be useful in reducing the risk of dementia among older adults.

## Background

Dementia is a major public health concern worldwide and is a leading contributor to disability and need for care among older adults**.** The cost of care of people with dementia has an enormous economic impact on the health care and social services system. In 2010, more than 35 million people worldwide were affected with dementia [1]. The Alzheimer Disease International estimated the total worldwide cost of dementia was US$604 billion in 2010 [2]. Currently there is no effective treatment available to cure dementia, therefore, a large body of research has focused on identifying reliable risk or modifiable risk factors that can delay or prevent the onset of dementia [3, 4]. One of the most consistent risk factors of dementia is aging, in particular, advanced age. Population-based studies have consistently found that aging is a major risk factor for dementia, and show that the prevalence of dementia increases exponentially with age [5]. Lower education has also been identified as a risk factor for dementia [6, 7]. It has been used as a proxy for brain and cognitive reserve with a large number of studies supporting the association between higher brain/cognitive reserve and lower risk of dementia [7–10]. Higher occupational attainment has been highlighted as a potential protective factor against the development of dementia. Previous meta-analysis by Valenzuela and Sachdev found that those with low occupational attainment had a higher risk of dementia compared to those with high occupational attainment [11]. The relationship between ethnicity and dementia is mixed, with some studies suggesting that the risk of dementia is higher in African American and Hispanic ethnicities compared to European American ethnicity [12, 13].

There is evidence suggesting that health factors such as vascular risk factors, depression, lifestyle factors such as physical activity, social relationships and educational attainment are important modifiable risk and protective factors of dementia [2, 3, 14]. Vascular risk factors such as cardiovascular diseases, stroke, diabetes, hypertension and obesity have been associated with increased risk of dementia [15–19]. For instance, Hayden and colleagues found that older adults with hypertension and stroke have a higher risk of developing vascular dementia [19]. Growing evidence links depression with dementia and suggests that the timing of depression may be important in defining the nature of this association [20]. It has been suggested that depression may be a risk factor for dementia or a prodromal phase of dementia [20–22]. A previous meta-analysis found that late-life depression increased the risk of incident all-cause dementia, in particular of both vascular dementia and Alzheimer’s disease (AD) [23]. Exercise and physical activity have been established as key modifiable lifestyle factors that protect against cognitive decline and dementia [24–27]. It was reported that regular physical activity has been associated with lower risks of all-cause dementia [24, 28]. The role of social relationship factors (i.e., social networks, social participation, frequency of social contact and loneliness) as a protective factor in the development of dementia has been supported with a number of longitudinal studies [3, 29, 30]. Meta-analysis conducted by Kuiper et al. has shown that lower frequency of social contact was associated with a higher risk of developing dementia compared to individuals with higher frequency of social contact [3]. Similarly, other potentially important social factors such as lower social participation and loneliness have been shown in meta-analyses to be associated with an increased risk of developing dementia.

To date, most published studies have come from Western populations which limit their generalizability to Asian population given the cultural differences. Singapore is a city-state country in Southeast Asia with a population of approximately 5.5 million [31]. It has a multiethnic urban population comprising Chinese, Malays and Indians as well as those belonging to other ethnicities. A recent study in Singapore [32] examined the risk factors of dementia and identified that older age, lower education, homemaker and retired status, and a history of stroke diagnosis were associated with a higher risk of dementia. Although various studies have demonstrated possible modifiable health and lifestyle risk factors of dementia, few studies have examined the pathways of how these factors influence each other as mediators and moderators that subsequently lead to dementia. Our study aimed to fulfill this research gap by investigating the pathways of how socio-demographic factors (age, gender, ethnicity, marital status, employment, and education), modifiable health factors including vascular risk factors (heart problem, stroke, transient ischaemic attack (TIA), diabetes, and hypertension), depression, and lifestyle risk factors (physical activity, social contact and loneliness) influence each other, and subsequently, lead to dementia. In addition, we hypothesized that modifiable factors such as vascular risk factors, depression, and lifestyle risk factors mediated the relationship between socio-demographic factors and dementia in a sample of Asian older adults.

## Methods

### Setting and study design

We used data from the Well-being of the Singapore Elderly (WiSE) study. The WiSE study was a nationally representative survey of the older adult population aged 60 years and above in Singapore. The sample was derived using a disproportionate stratified sampling design. We oversampled residents aged 75 years and above, and those of Malay and Indian ethnicity in order to ensure that sufficient sample size for these population subgroups could be achieved to improve the reliability of our estimates. The study was approved by the institutional ethics review boards of participating institutions which include the National Healthcare Group, Domain Specific Review Board and the SingHealth Centralised Institutional Review Board. Written informed consent was obtained from all respondents. In the case where respondents were unable to provide informed consent, written informed consent was taken from their legally acceptable representative/next of kin. The respondents were randomly selected from a national registry that maintains the names, other socio-demographic details such as age, gender and ethnicity, and household addresses of all residents in Singapore. The respondents were approached at the household address provided by the registry. Face-to-face interviews were conducted by professional survey interviewers at the residence of the older adults using an online Computer Assisted Personal Interviewing application and data were collected in real-time. We had anticipated that participants may be concerned about the confidentiality of their responses especially since the interview asks certain sensitive questions and generates psychiatric diagnoses. Therefore, all interviewers underwent a 2-week training that included ethical guidelines and the administration of study questionnaires to ensure that they administered the questionnaires sensitively and appropriately. Interviewers were encouraged to suggest alternate times or sites such as the void decks in housing estates which are generally quiet during the daytime if they felt that there was no privacy in the older adults home. The collection of research data without participant’s identifiers such as name, and other identifiable data further ensured confidentiality**.** The study method has been described in detail elsewhere [32].

### Measures

Dementia diagnosis was established according to 10/66 criteria [33]. The 10/66 dementia diagnosis algorithm [34] requires: a structured clinical mental state interview, the Geriatric Mental State (GMS), which applies the Automated Geriatric Examination for Computer Assisted Taxonomy (AGECAT) computer algorithm [35]; a cognitive test battery comprising the Community Screening Instrument for Dementia (CSI-D) [36] which incorporated the Consortium to Establish a Registry for Alzheimer’s Dementia (CERAD) animal naming verbal fluency task and modified CERAD 10 word list learning task with delayed recall to generate the global cognitive score (COGSCORE) and item-weighted total score from the participant cognitive test [37] and; an informant interview, the CSI-D informant score (RELSCORE) [36] for evidence of cognitive and functional decline. The data were gathered from both participants and their caregivers to generate dementia diagnosis. The dementia diagnosis was given to respondents scoring above a cut-point of predicted probability of dementia derived from the logistic regression equation developed in the 10/66 international pilot study, using coefficients from the GMS, CSI-D and 10 word list learning tasks [34]. Its algorithm has been cross-culturally validated elsewhere [33, 34, 38] and in our sample [32]. Data on other recorded health conditions including hypertension, TIA, depression, heart problem (myocardial infarction, cardiac failure and valvular heart disease), stroke and diabetes as well as socio-demographic characteristics including age, gender, ethnicity, marital status, education, and employment were also collected. Social relationship factors such as frequency of contact were assessed by three items asking respondents on the frequency of contact with children or other relatives, friends and neighbors (e.g. “How often do you see any of your children or other relatives to speak to?”). Six response options were available for these 3 items. For the purpose of analysis, “Never”, “At least monthly” and “Less often” responses were combined and recoded as ‘Less frequent contact’. While “Daily”, “2–3 times weekly” and “At least weekly” responses were recoded as ‘More frequent contact’; reducing the number of categories from six to two. Loneliness was measured by a single item, “Do you ever feel lonely?” with 3 response alternatives: “Yes, often”, “Yes, sometimes”, and “No, never”. With regards to physical activity, participants were asked: “Taking into account both work and leisure, would you say that you are very physically active, fairly physically active, not very physically active or not at all physically active?”. The responses “Very physically active” and “Fairly physically active” were combined and recoded as “Physically active”, while “Not very physically active” and “Not at all physically active” were combined and recoded as “Not physically active”.

### Statistical analysis

Statistical analyses were carried out using the SAS software version 9.2 (SAS Institute Inc., Cary, NC, USA) and Mplus version 5.21. To ensure that the survey findings were representative of the Singapore older adult population, all estimates were analyzed using survey weights to adjust for oversampling, non-response and post stratification according to age and ethnicity of the Singapore older adult population. Mean and standard errors were calculated for continuous variables, and frequencies and percentages for categorical variables.

**Fig. 1:**
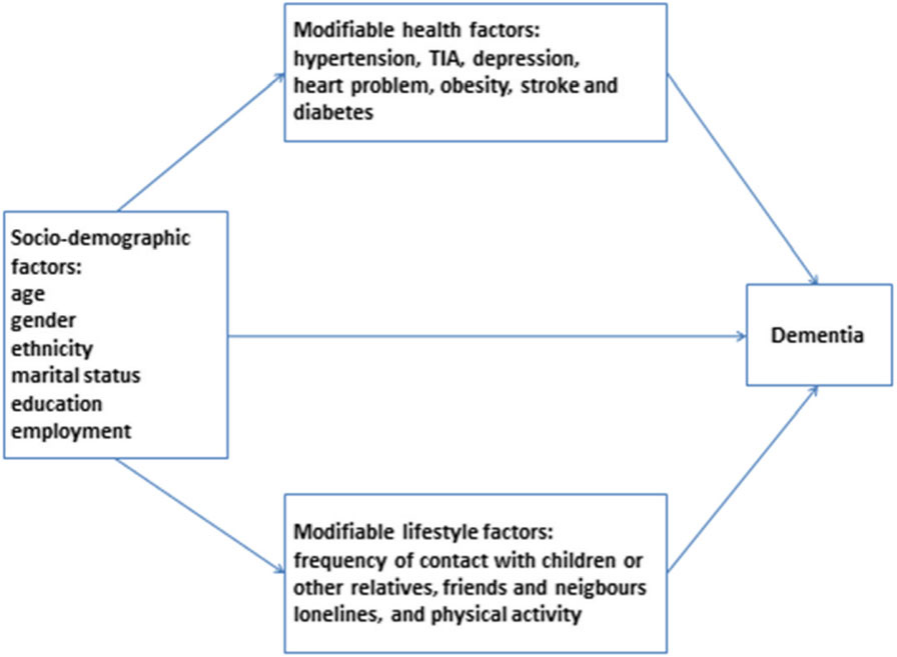
Hypothesized pathways associations between socio-demographic, modifiable health and lifestyle factors and dementia

Structural equation modelling (SEM) without latent variables was applied to confirm the hypothesized mediation model that examined the pathways of how socio-demographic factors, modifiable health factors and lifestyle factors influence each other, and subsequently, lead to dementia. Figure 1 presents the initial hypothesized pathways model of the relationship between socio-demographic factors, modifiable health factors, lifestyle factors and dementia, which was derived from the literature. We used a two-step strategy to analyse the data [39]. In the first step, we designed the hypothesized pathways model as shown in Fig. 1 and fitted with our older adult general population sample. The misspecification of the initial model was examined using Modification Indices (MI). MI was used to indicate how much the overall model chi-square decreases or improves if a constrained parameter is freely estimated. Candidate paths involving conspicuous values (MI > 10) were then examined for the actual amount of model fit improvement and for the magnitude of the ensuing freely estimated coefficients. The decision to explore and keep new pathways also followed their theoretical meaningfulness. The second step involved systematically trimming out non-significant pathways, i.e. coefficient estimates with *p*-value >0.05. This process adhered to the hierarchical principal advocated in the model. The trimming process thus moved from left to right of Fig. 1. At each step, interim evaluations of MI were carried out in search of any relevant pathways arising once the model had been simplified. The overall process stopped when no additional significant pathways were suggested by the MI, while all remaining pathways retained statistical significance given acceptable levels of model fit. In this study, direct effect of categorical predictor variable on binary outcome variable as well as indirect effect indicated by the effect of the categorical predictor on binary outcome variable via binary mediator variable were defined using model constraint procedure via maximum likelihood robust estimator. Total effects denoted as the sum of direct effects and indirect effects were also calculated [40]. The significance of the indirect effects were obtained using Monte Carlo simulation method [40]. The SEM analyses employed Mplus’ robust weighted least squares mean and variance adjusted estimator (WLSMV) to include categorical and non-normal variables [41]. Goodness-of-fit was mainly evaluated using three indices. The Root Mean Square Error of Approximation (RMSEA) incorporates a penalty function for poor model parsimony, values under 0.06 suggest close approximate (adequate) fit, whereas values above 0.10 indicate poor fit and that the model should be rejected [42]. The Comparative Fit Index (CFI) and the Tucker-Lewis index (TLI) represent incremental fit indices contrasting the hypothesized model to a more restricted nested baseline model, the “null model”. Both range from zero to one and values >0.95 are indicative of adequate fit [42].

## Results

### Socio-demographic characteristics of the sample

A total of 2565 respondents completed the study giving a response rate of 65.6%. Informants of 2421 respondents completed the requisite interviews and data of these respondents and informants were included in this study. The sample comprised 57% female and 43% male respondents. Majority of the respondents were aged between 60 and 74 years (74.8%), of Chinese ethnicity (82.6%), and currently married (65.4%) (Table 1). The prevalence of dementia in this study was 10% [32]). The percentages of heart problem, stroke, diabetes, hypertension, TIA and depression were 11.9%, 7.6%, 26.4%, 74.1% [43], 1.9% and 8.3%, respectively. Majority of the respondents were physically active (75%), had ‘more frequent contact’ with their children or other relatives (83.9%), friends (85.6%) and neighbors (53.5%).

**Table 1:** Socio-demographic characteristics of the sample

	Unweighted N	Unweighted %	Weighted %
Overall	2421	100	100
Age group			
60–74	1403	58.0	74.8
75–84	633	26.2	19.4
85+	385	15.9	5.7
Gender			
Men	1039	42.9	43.0
Women	1382	57.1	57.0
Ethnicity			
Chinese	931	38.5	82.6
Malay	728	30.1	9.8
Indian	728	30.1	6.1
Others	34	1.4	1.5
Marital status			
Never married	108	4.5	6.8
Married/cohabiting	1419	58.7	65.4
Widowed	798	33.0	22.8
Divorced/separated	94	3.9	5.0
Education			
None	502	20.9	17.1
Some, but did not complete primary	579	24.1	23.8
Completed primary	597	24.8	24.1
Completed secondary	488	20.3	22.5
Completed tertiary	241	10.0	12.5
Employment status			
Paid work (part-time and full-time)	632	26.4	32.9
Unemployed	30	1.3	1.4
Homemaker	782	32.7	27.2
Retired	947	39.6	38.5

### Final pathways model

**Fig. 2:**
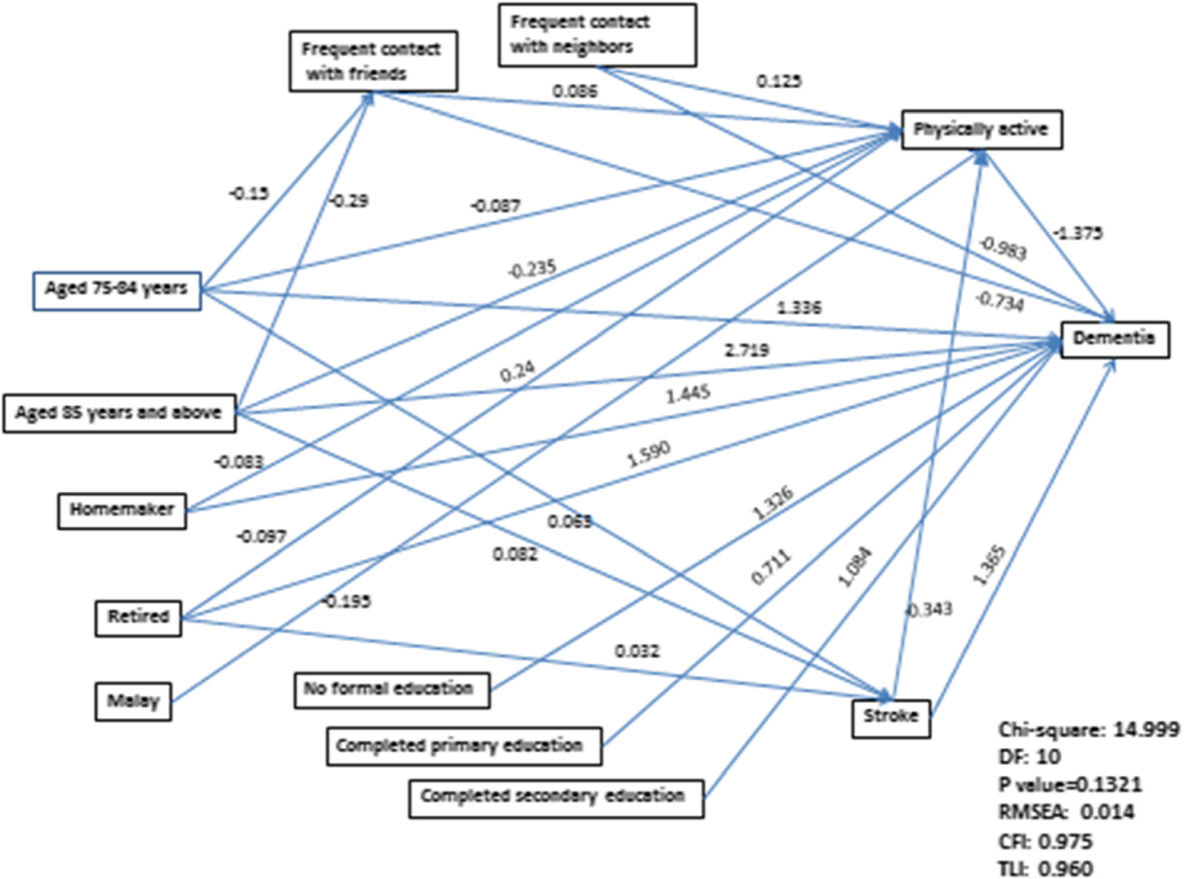
Final pathways model. Legend: A single-headed arrow represents a direct effect of one variable on another. Unstandardized logistic regression coefficient are presented. DF: degree of freedom. RMSEA: Root Mean Square Error of Approximation. CFI: Comparative Fit Index. TLI: Tuker-Lewis index

Figure 2 shows significant pathways of the final model and their goodness-of-fit indices. The measures of model fit were as follows: chi-square of model fit (χ^2^ = 14.999, *df* = 10, *p* = 0.1321), TLI (0.960), CFI (0.975) and RMSEA (0.014). All indices suggest that the final model fit the data well. The final model shows that those aged 75–84 years (OR =3.8, 95% CI = 2.2, 6.5) and 85 years and over (OR =15.3, 95% CI = 8.8, 26.3) (vs. 60–74 years), having no formal education (OR =3.8, 95% CI = 2.0, 7.1), completed primary education (OR =2.0, 95% CI = 1.03,4.0), completed secondary education (OR =3, 95% CI = 1.5, 5.7) (vs. completed tertiary), homemaker (OR =4.2, 95% CI = 1.9, 9.6) and retired (OR =4.9, 95% CI = 2.2, 11.1) (vs. paid work), and had a history of stroke (OR =3.9, 95% CI = 2.2, 7.0) were directly associated with higher odds of having dementia, while those who had more frequent contact with friends (OR =0.5, 95% CI = 0.3, 0.8) and neighbors (OR =0.4, 95% CI = 0.2, 0.6) and were physically active (OR =0.3, 95% CI = 0.2, 0.4) were directly associated with lower odds of having dementia.

We also found that physical activity, more frequent contact with friends and stroke played a significant role as mediators in these relationships. Physical activity significantly mediated the relationship between those aged 75–84 years (OR = 1.1, 95% CI = 1.02, 1.2) and 85 years and over (OR =1.4, 95% CI = 1.2, 1.6), homemakers (OR =1.1, 95% CI = 1.02, 1.2) and retired (OR =1.1, 95% CI = 1.05, 1.2), had more frequent contact with friends (OR =0.9, 95% CI = 0.8, 0.9) and neighbors (OR =0.8, 95% CI = 0.8, 0.9), stroke (OR =1.6, 95% CI = 1.3, 1.9) and Malay ethnicity (OR =1.3, 95% CI = 1.2, 1.4) with dementia. More frequent contact with friends significantly mediated the relationship between those aged 75–84 years (OR =1.1, 95% CI = 1.01, 1.2), 85 years and over (OR =1.2, 95% CI = 1.03, 1.9) and dementia, while stroke significantly mediated the relationship between those aged 75–84 years (OR = 1.11, 95% CI = 1.01,1.3) and 85 years and over (OR = 1.1, 95% CI = 1.02,1.3) and dementia. The overall path model explained 57.7% of the variance in dementia.

## Discussion

The current study examined the extent to which socio-demographic, health and lifestyle factors influence each other, and subsequently, lead to dementia. The study was analyzed using a SEM approach using data from a nationally representative Singapore older adult sample. The final pathways model indicated that the model had good fit indices. Primary findings show that those of older age, low educational attainment, homemaker and retired status, with a history of stroke, having more frequent contact with friends and neighbors and being physically active were directly and indirectly associated with dementia. The study also found that physical activity, stroke and more frequent contact with friends played an important role as mediators in the final pathways model. These findings suggest that older adults who initially demonstrated greater risk of dementia were significantly less likely to have dementia if they were physically active and had frequent contact with friends. This study confirms findings from previous studies [24, 25, 29, 30] which showed that physical activity and more frequent contact friends were protective against dementia.

The relationship between physical activity and brain structure has been reported in numerous epidemiological and observational studies. According to a review paper by Rolland et al. [14], 20 out of 24 longitudinal human epidemiological studies found that physical activity was associated with a reduced risk for cognitive decline and dementia. In a cohort study of 4761 older adults, Chang et al. [44] showed that midlife physical activity was associated with reduced prevalence of dementia and cognitive decline 26 years later. Boyle et al. [45] suggest that physical activity may influence brain structure and reduce AD risk through multiple physiological mechanisms. They may be broadly grouped into three categories: (i) counteraction of AD pathology, such as anti-amyloid effects, (ii) influencing the levels of neurotrophic factors and neurotransmitters, that may optimize neuronal function, and (iii) reducing vascular risk factors that can independently compromise brain structure.

Our findings demonstrate that having more frequent contact with friends and neighbors is associated with lower risk of dementia. The importance of social contact in dementia is consistent with a recent meta-analysis. Kuiper et al. [3] categorized social relationship factors into (1) social network size; (2) social participation (e.g., participation in associations or community activities); (3) frequency of social contact (e.g., visiting or receiving phone calls from friends, children or other relatives) and found that individuals with higher frequency of social contact had a lower risk of developing dementia. One potential explanation of why more social interaction such as high frequency of social contact protects against dementia relates to the ‘use it or lose it’ theory, which suggests that engagement in intellectual, social and physical activities stimulates the brain [3]. The ‘use it or lose it’ theory is related to the cognitive reserve theory which suggests that social interaction affects brain structure and results in more efficient use of brain networks [46].

A significant direct effect of stroke on dementia is also consistent with previous findings in the literature [15–17]. We found that physical activity mediated the relationship between stroke and dementia. These results suggest that older adults with a history of stroke and those less likely to be physically active had a higher risk of dementia. In concordance with previous studies [6–11], we found that high risk of dementia was influenced by lower educational attainment such as no formal education, completed primary education and secondary education (vs. completed tertiary) and lower occupational attainment such as homemaker and retired (vs. employed). This finding is consistent with a meta-analysis by Valenzuela and Sachdev [11] which reported that those with low educational and occupational attainment had a higher risk of dementia compared to those with higher educational and occupational attainment. Higher educational and occupational attainments have been highlighted as potentially protective factors against the risk of developing dementia. Cognitive reserve has become the dominant explanatory framework between education and dementia, to the point where education is now widely used as a proxy for cognitive reserve [2, 46]. Our study adds new findings to show that the relationship between occupation and dementia was mediated by physical activity. This is also consistent with the ‘cognitive reserve’ theory, which suggests that engagement in intellectual, social and physical activities (via occupational attainment and higher level of physical activity) may stimulate the brain and result in more efficient use of brain networks, buffering the effects of neuropathology on cognition [46].

The current findings rules out depression as a predictor of dementia and mediator for the relationship between sociodemographic factors and dementia. While the current finding is consistent with our previous finding that found depression was not significantly associated with dementia in multivariate analysis [32], we can only speculate regarding this non-significant finding. It is possible that the effect of depression is reduced after adding multiple mediators in the final pathways model. For example, in standard simple logistic regression analysis, we found depression was significantly associated with a 2-fold increased risk of dementia (OR = 2.2, 95% CI = 1.4, 3.6). However, after controlling for other mediator variables such as stroke, physical and frequency contact with friends and neighbors in the multivariate logistic regression analysis, the effect size of depression was reduced to 30% (OR = 1.5, 95% CI = 0.9,2.7). It is possible that these variables may alter the relationship between depression and dementia since previous studies have found that exercise could reduce depressive symptoms among older adults with and without dementia [47–49]. Thus, future studies are needed to explore the role of exercise and social contact as mediators/moderators for the relationship between sociodemographic factors and depression and relationship between depression and dementia.

There is a possibility that the links between social connectedness, depression and dementia might be reciprocal. A reciprocal relationship between loneliness and cognitive function, and prospective loneliness-cognition relationship being partially mediated by physical health has been reported in a 9-year cross-lagged longitudinal data in Chinese older adults [50]. Given that our results are based on cross-sectional data we are unable to explore whether there are significant reciprocal relationships over time between social connectedness, depression and dementia in our data. Future longitudinal studies are needed to address this issue.

Limitations to the present study should be noted while interpreting the results. Firstly, although we were able to quantify the mediating role of physical activity, more frequent contact with friends and stroke in our final pathways model, the cross-sectional data limits our ability to draw conclusions about the causality of the observed relationship. Secondly, the effect of recall bias cannot be excluded in this study. Notwithstanding these limitations, this is a nationwide population-based survey, representative of a multi-ethnic older adult population in Singapore with a large sample size. The study was a single phase assessment, using widely accepted assessments and questionnaires with face-to-face interviews which ensured accurate and detailed collection of information from all individuals. We used SEM models because these models are expected to provide more insight into the relative importance of the multiple predictors and mediators of dementia. The SEM models allowed us to analyze simultaneously the direct and indirect relationships of the modifiable factors and mediators with dementia.

## Conclusions

In conclusion, our results suggest that physical activity, social contact and stroke were potential mediators in the relationship between age, education, employment and dementia. Intervention programs focusing on physical activity such as exercise and social contact may be useful in preventing dementia among older adults.
